# Efficacy and Immune Correlates of OMP-1B and VirB2-4 Vaccines for Protection of Dogs from Tick Transmission of Ehrlichia chaffeensis

**DOI:** 10.1128/mbio.02140-22

**Published:** 2022-11-07

**Authors:** Khemraj Budachetri, Mingqun Lin, Rory C. Chien, Wenqing Zhang, Guy Nathaniel Brock, Yasuko Rikihisa

**Affiliations:** a Department of Veterinary Biosciences, College of Veterinary Medicine, Infectious Diseases Institute, Ohio State University, Columbus, Ohio, USA; b Center for Biostatistics, Ohio State University, Columbus, Ohio, USA; Iowa State University

**Keywords:** *Ehrlichia*, OMP-1B, VirB2-4, vaccine, dog, tick transmission, ISCOM, IFN-γ, in-tick neutralization

## Abstract

Ehrlichia chaffeensis, an obligatory intracellular bacterium, causes human monocytic ehrlichiosis, an emerging disease transmitted by the Lone Star tick, Amblyomma americanum. Here, we investigated the vaccine potential of OMP-1B and VirB2-4. Among the highly expressed and immunodominant *E. chaffeensis* porin P28s/OMP-1s, OMP-1B is predominantly expressed by *E. chaffeensis* in *A. americanum* ticks, whereas VirB2-4 is a pilus protein of the type IV secretion system essential for *E. chaffeensis* infection of host cells. Immunization with recombinant OMP-1B (rOMP-1B) or recombinant VirB2-4 (rVirB2-4) protected mice from *E. chaffeensis* infection as effectively as Entry-triggering protein of *Ehrlichia* immunization. Dogs vaccinated with a nanoparticle vaccine composed of rOMP-1B or rVirB2-4 and an immunostimulating complex developed high antibody titers against the respective antigen. Upon challenge with *E. chaffeensis-*infected *A. americanum* ticks, *E. chaffeensis* was undetectable in the blood of rOMP-1B or rVirB2-4 immunized dogs on day 3 or 6 post-tick attachment and for the duration of the experiment, whereas dogs sham-vaccinated with the complex alone were persistently infected for the duration of the experiment. *E. chaffeensis* exponentially replicates in blood-feeding ticks to facilitate transmission. Previously infected ticks removed from OMP-1B-immunized dogs showed significantly lower bacterial load relative to ticks removed from sham-immunized dogs, suggesting in-tick neutralization. Peripheral blood leukocytes from rVirB2-4-vaccinated dogs secreted significantly elevated amounts of interferon-γ soon after tick attachment by ELISpot assay and reverse transcription-quantitative PCR, suggesting interferon-γ-mediated *Ehrlichia* inhibition. Thus, *Ehrlichia* surface-exposed proteins OMP-1B and VirB2-4 represent new potential vaccine candidates for blocking tick-borne ehrlichial transmission.

## INTRODUCTION

The incidence of tick-borne diseases has risen dramatically in the past 2 decades and continues to rise (1, 2). Human monocytic ehrlichiosis (HME) is one of the most prevalent life-threatening emerging tick-borne zoonoses in the United States (3–5). Depending on geographic location and risk of tick bite, HME incidence rates as high as 660 per 100,000 population have been reported (6–8). HME is caused by Ehrlichia chaffeensis, an obligatory intracellular bacterium of the order Rickettsiales. *E. chaffeensis* replicates within human blood monocytes and causes severe systemic flu-like symptoms (3). Delayed therapy initiation due to misdiagnosis, underlying illness or injury, stress, immunosuppression, and/or co-infection with other tick-borne pathogens, for example, can lead to severe complications or death (mortality rate of 2% to 5%) (3). Infection of humans with *Ehrlichia* and closely related pathogens has also occurred through blood transfusion (9, 10). Treatment options are limited to the broad-spectrum antibiotic doxycycline and no vaccine is currently available (11).

The Lone Star tick (Amblyomma americanum) serves as the primary biological vector for *E. chaffeensis* (1). Its range across the US is extensive—from Texas to central Wisconsin in the Midwest and east to the coast, where it can be found as far north as Maine (12, 13). As the tick continues to expand its range, the geographic range of risk for *E. chaffeensis* infections will likely expand (14). White-tailed deer (Odocoileus virginianus) are the well-known natural reservoirs for *E. chaffeensis* (15, 16), in addition to serving as important hosts to all three mobile stages of the Lone Star tick (17). White-tailed deer have overpopulated much of the US in recent years, contributing to the emergence and expansion of HME (1).

Numerous obstacles exist in developing a vaccine against *E. chaffeensis*, including the dearth of knowledge on the *E. chaffeensis* antigens which stimulate protective immunity. Thus, we researched *E. chaffeensis* immunodominant proteins (i.e., recognized by human immune system), *E. chaffeensis* surface exposed proteins (i.e., accessible by antibodies), and functional proteins required for *E. chaffeensis* survival (i.e., lethal target) which lack homology to human proteins. Our first HME vaccine candidate is the C terminus of Entry-triggering protein of *Ehrlichia* EtpE (EtpE-C), an outer membrane invasin that directly binds to the *E. chaffeensis* receptor (DNase X) on mammalian host macrophages and triggers *E. chaffeensis* endocytosis (18, 19). Dogs are quite susceptible to tick-borne pathogens, including *E. chaffeensis* (20, 21), and thus can serve as sentinels; importantly, dog infection poses a risk of transmission to humans. We reported that dogs vaccinated with a nanoparticle vaccine composed of recombinant EtpE-C (rEtpE-C) and immunostimulating complexes (ISCOMs) developed *E. chaffeensis*-neutralizing antibodies and interferon (IFN)-γ responses and showed significantly accelerated *E. chaffeensis* clearance upon *E. chaffeensis* infection by experimentally infected ticks (22). However, EtpE-C vaccination did not prevent *E. chaffeensis* transmission from infected ticks to dogs (22).

In this study, we examined the potential of two other *E. chaffeensis* outer membrane proteins (OMP-1B and VirB2) as HME vaccines. P28/OMP-1 proteins are among the most highly expressed and immunodominant *E. chaffeensis* proteins (23). *E. chaffeensis* P28s/OMP-1s function as porins which allow nutrient diffusion across the outer membrane of bacteria (24). We also showed (23), and others independently confirmed (25, 26), that immunizing mice with recombinant P28 (also called P28-19, OMP-1g) protects them against intraperitoneal challenge with *E. chaffeensis* cultured in mammalian macrophages. Furthermore, monoclonal antibodies against P28 (OMP-1g) protect severe-combined immunodeficiency mice against fatal infection with *E. chaffeensis* (27). Whereas multiple *p28s/omp-1s* are expressed by *E. chaffeensis* in mammalian cells and dogs, *omp-1B* is the only *E. chaffeensis p28/omp-1* gene expressed in infected *A. americanum* ticks (28), and OMP-1B (also called p28-Omp14 [29]) protein is the only OMP-1/P28 paralog detected in *E. chaffeensis* cultured in the tick cell line ISE6, as shown by proteomics (29). *p28* expression in human monocytes (THP-1) and *omp-1B* expression by *E. chaffeensis* in the *Amblyomma* and *Ixodes* tick cell lines AAE2 and ISE6, respectively, were also detected in the respective transcriptomes by microarray-based analyses (30). Thus, we tested OMP-1B as a second vaccine candidate for preventing tick transmission of *E. chaffeensis*.

The third vaccine candidate is the highly expressed *E. chaffeensis* surface protein VirB2, which is a Type IV secretion system (T4SS) pilus protein required for secretion of at least three distinct T4SS effector molecules into the host cell cytoplasm (31). Secreted T4SS effectors (i) induce host cell autophagy, enabling *E. chaffeensis* to acquire catabolites as nutrients while blocking host cell apoptosis and reactive oxygen species generation; (ii) induce ferritinophagy to competitively acquire intracellular iron as a nutrient; and (iii) block lysosomal fusion with *E. chaffeensis*-containing vacuoles (32–39). Thus, VirB2 is expected to be essential for *E. chaffeensis* survival and replication.

Dogs are naturally infected with *E. chaffeensis* (21, 40, 41) and can serve as a useful animal model for tick transmission of *E. chaffeensis* (28, 42). However, exposing a large number of specific pathogen-free (SPF) dogs to *E. chaffeensis*-infected ticks while in the care of a biosafety level 2 (BSL-2) facility is costly, labor-intensive, and space-prohibitive, as well as being an animal welfare concern. Thus, we first tested OMP-1B and VirB2-4 (the highest-expressed VirB2 paralog based on our global proteomics [43]) vaccines in immunocompetent mouse infection model (23) using the previously proven protective antigen EtpE-C (19, 22) as a positive control. Next, we examined whether vaccinating dogs with recombinant OMP-1B (rOMP-1B) and recombinant VirB2-4 (rVirB2-4) can prevent tick-mediated transmission of *E. chaffeensis* to dogs, and potential immune correlates of protection.

## RESULTS

### Vaccination of mice with rOMP-1B and rVirB2-4 curtails *E. chaffeensis* infection.

**FIG 1:**
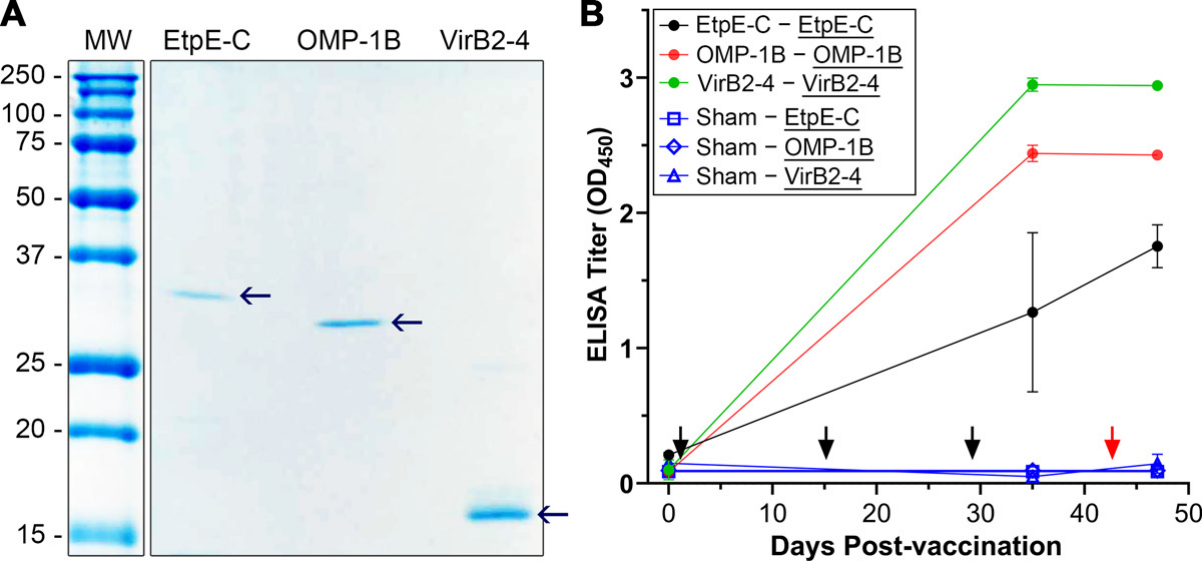
Mice vaccinated with recombinant EtpE-C (rEtpE-C), recombinant OMP-1B (rOMP-1B), or recombinant VirB2-4 (rVirB2-4) develop high antibody titers against the respective antigen. (A) Recombinant proteins (40 ng each of rEtpE-C, rOMP-1B, and rVirB2-4) were subjected to SDS-PAGE and GelCode Blue staining. Molecular size: rEtpE-C, 31 kDa; rOMP-1B, 28 kDa; and rVirB2-4, 15 kDa. The molecular weight (MW) markers are shown in kilodaltons. (B) Enzyme-linked immunosorbent assay (ELISA) titers using the three recombinant proteins as the antigen (underlined). Filled circles, mice vaccinated with rEtpE-C (black), rOMP-1B (red), and rVirB2-4 (green); Sham, sham-vaccinated mice (open circles). Results are shown as means ± standard deviation (SD) from three vaccinated and three sham-vaccinated mice. Black arrows indicate days on which mice were vaccinated, red arrow denotes the day on which mice were challenged with *E. chaffeensis*.

**FIG 2:**
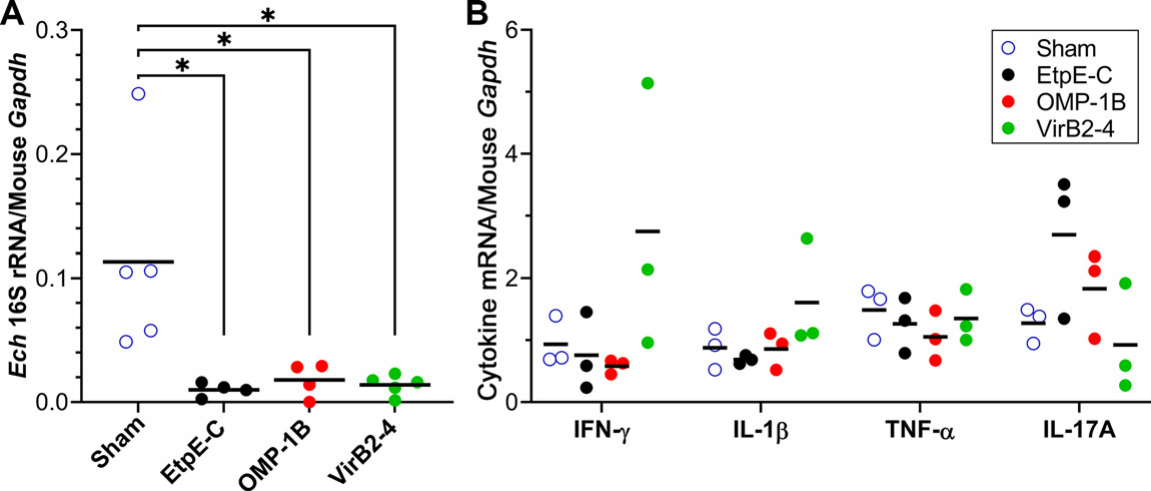
rOMP-1B or rVirB2-4 immunization protects mice from Ehrlichia chaffeensis infection as effectively as rEtpE-C immunization with low cytokine responses. (A) Relative *E. chaffeensis* (*Ech*) 16S rRNA gene/mouse *GADPH* levels in peripheral blood from three recombinant protein-vaccinated and sham-vaccinated mice 5 days after intraperitoneal challenge with *E. chaffeensis* (quantitative PCR [qPCR]). The difference between the vaccinated and sham-vaccinated mice was significant (*P < *0.05, *n* = 4 or 5) by one-way analysis of variance (ANOVA), whereas there were no significant differences among the three vaccines. (B) Mouse interferon (IFN)-γ, interleukin (IL)-1β, tumor necrosis factor (TNF)-α, and IL-17A gene expression was estimated in spleen samples from sham-vaccinated and recombinant protein-vaccinated mice at 5 days after intraperitoneal challenge with *E. chaffeensis* using gene-specific primers; the data were normalized using mouse GAPDH (glyceraldehyde 3-phosphate dehydrogenase) (RT-qPCR). Horizontal bars indicate mean values. Cytokine expression was not significantly different between vaccinated and sham-vaccinated mice based on a one-way ANOVA (*n* = 3).

Newly cloned *E. chaffeensis omp1-B* and *virB2-4*, along with previously cloned *etpE-C* (the fragment of the gene encoding C terminus of EtpE) (18), were expressed in E. coli and recombinant proteins were cobalt affinity-purified to homogeneity (Fig. 1A). Immunization of mice with rOMP-1B, rVirB2-4, or rEtpE-C with Quil A adjuvant induced specific antibodies against each immunogen (Fig. 1B). At 14 days after the last vaccination, mice were intraperitoneally challenged with *E. chaffeensis* and clinical signs were monitored. All mice were euthanized at day 5 post-challenge to determine *E. chaffeensis* infection by quantitative PCR (qPCR) in the blood specimens. As shown in Fig. 2A, rVirB2-4 or rOMP-1B immunization was as effective as rEtpE-C with respect to lowering *E. chaffeensis* infection in mice. The production of mRNA encoding IFN-γ and other cytokines (interleukin [IL]-1β, tumor necrosis factor [TNF]-α, and IL-17A) in spleen samples from *E. chaffeensis*-challenged mice was measured by reverse transcription-quantitative PCR (RT-qPCR). None of these cytokines were significantly elevated in these mice compared to sham-immunized mice, although there was a slight elevation of IFN-γ mRNA in rVirB2-4-immunized mice and a slight elevation of IL-17A mRNA in rEtpE-C-immunized mice (Fig. 2B). Mice did not show any clinical signs (weight loss, lethargy, anorexia, squinting eyes, or ruffled fur) throughout experiments.

### OMP-1B and VirB2-4 are expressed by *E. chaffeensis* in adult *A. americanum* ticks needle-injected as nymphs.

**FIG 3:**
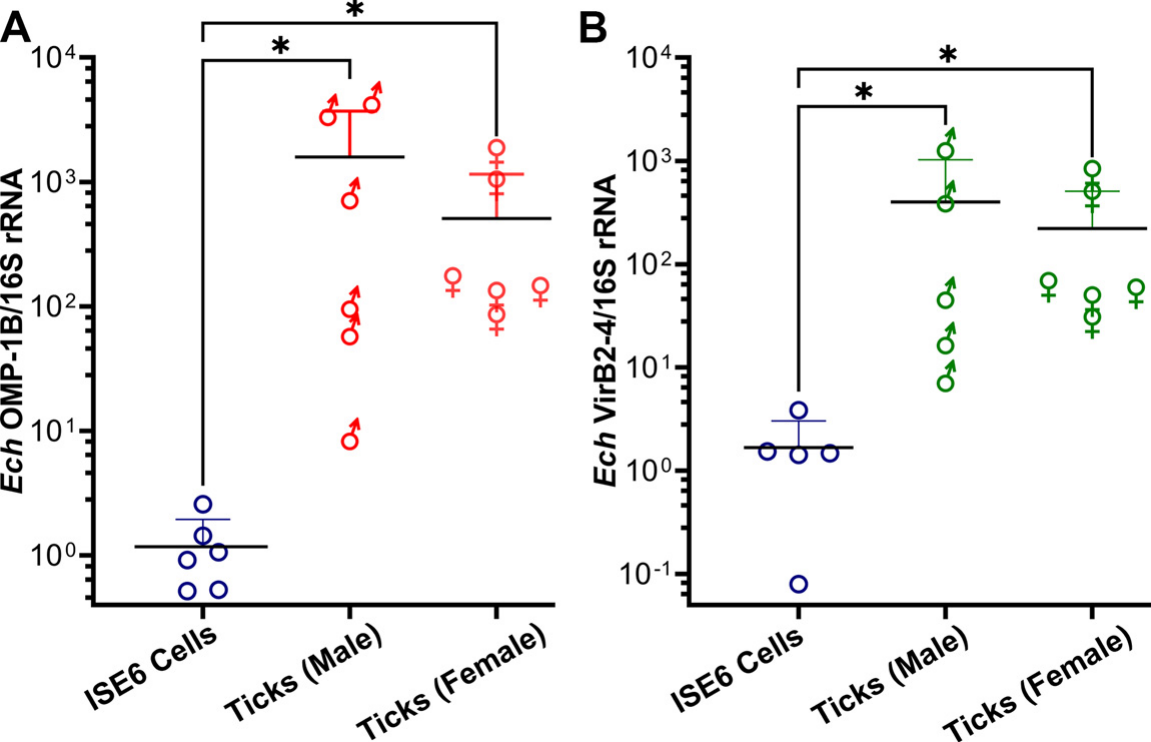
OMP-1B and VirB2-4 are expressed by *E. chaffeensis* in adult Amblyomma americanum ticks infected as nymphs. (A and B) Expression of OMP-1B mRNA (A) and VirB2-4 mRNA (B) by *E. chaffeensis* (*Ech*) in ISE6 tick cells and in molted, unfed male and female *A. americanum* ticks normalized by *E. chaffeensis* 16S rRNA (RT-qPCR). *, *P < *0.05; one-way ANOVA. Horizontal bars indicate mean values.

*A. americanum* are three-host ticks, as they consume their first and second blood meals from different hosts during the larval and nymphal stages, respectively, to molt into the adult stage, and take their third meal to mate and lay eggs (44). Given the effectiveness of the rOMP-1B and rVirB2-4 vaccines in mice, we prepared *E. chaffeensis*-infected ticks for our experimental transmission study in dogs. For rOMP-1B and rVirB2-4 vaccine studies, 400 and 370 freshly engorged *A. americanum* nymphs, respectively, were needle-injected with *E. chaffeensis* (7 × 10^8^ to 10 × 10^8^ bacteria in 2 to 4 μL per tick) freshly isolated from infected DH82 cells. The injected nymphal ticks were allowed to molt in an incubator, resulting in 130 male and 260 female adult ticks (molting efficiency, 97.5%) and 122 male and 226 female adult ticks (molting efficiency, 94.1%) for rOMP-1B and rVirB2-4 study, respectively. *E. chaffeensis* infection in the molted adult ticks was verified by RT-qPCR of *E. chaffeensis* 16S rRNA. All tested ticks (*n* = 24; 12 females, 12 males) were infected with *E. chaffeensis*, indicating effective transstadial transmission in ticks. Expression of OMP-1B and VirB2-4 mRNA by *E. chaffeensis*, which was normalized to that of *E. chaffeensis* 16S rRNA, was significantly greater in both female and male ticks than *E. chaffeensis* in ISE6 tick cells, as assessed with RT-qPCR (Fig. 3A and B).

### Vaccination of dogs with rOMP-1B and rVirB2-4 induces antibodies against respective antigens and prevents *E. chaffeensis* transmission from infected ticks.

ISCOM is a spherical, open cage-like structure (30 to 40 nm in diameter) composed of cholesterol, phospholipids, and Quillaia saponins (45). The complex is used in vaccines because it induces a strong immune response with minimal side effects (46). SPF beagle dogs (ten dogs; five males and five females, aged 1 to 2 years) were vaccinated with rOMP-1B and ISCOM (one male and two females), rVirB2-4 and ISCOM (2 males and 1 female), or ISCOM alone (sham vaccination control; two males and two females) three times at 2-week intervals. Due to BSL-2 space constraints and other restrictions, we carried out two sequential experiments with five dogs each (three rOMP-1B and two sham-vaccinated dogs) and (three rVirB2-4 and two sham-vaccinated dogs). At 14 to 24 days after the last vaccination, 60 *E. chaffeensis*-infected ticks (40 females, 20 males) were allowed to feed on each dog.

**FIG 4:**
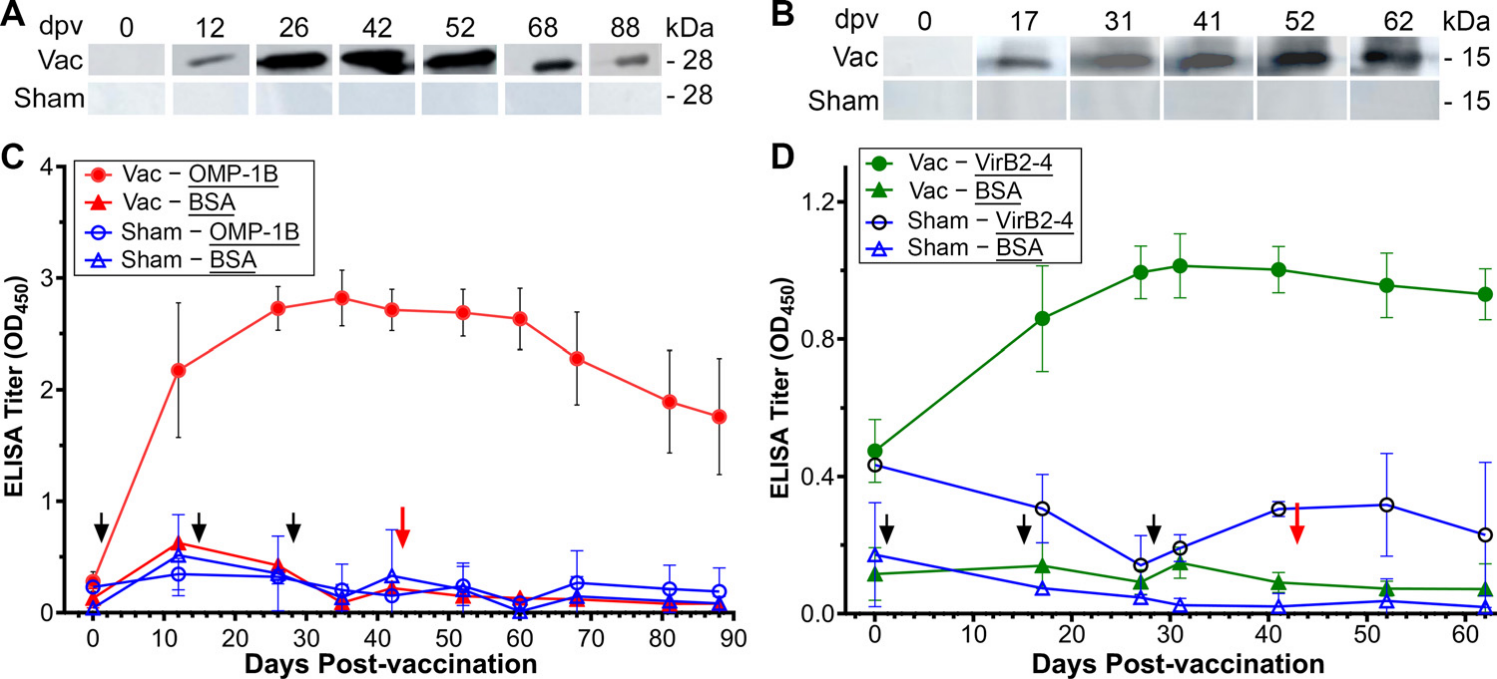
Dogs vaccinated with rOMP-1B or rVirB2-4 develop high antibody titers to their respective immunogens. (A and B) Western blotting for rOMP-1B (A) and rVirB2-4 (B) using plasma from a vaccinated dog (Vac) and a sham-vaccinated dog (Sham). Representative data are shown from one of three vaccinated or one of four sham-vaccinated dogs. dpv, days post-vaccination. (C and D) ELISA titers against rOMP-1B (C), rVirB2-4 (D), or bovine serum antigen (BSA; negative control, panels C and D) as the antigen (underlined). Data are shown for rOMP-1B-vaccinated (red, panel C), rVirB2-4-vaccinated (green, panel D), and sham-vaccinated (blue, panels C and D) dogs. Black arrows indicate days on which dogs were vaccinated, and red arrow denotes day of tick challenge. Data are shown as the means ± SD from three vaccinated dogs and four sham-vaccinated dogs for each condition.

**FIG 5:**
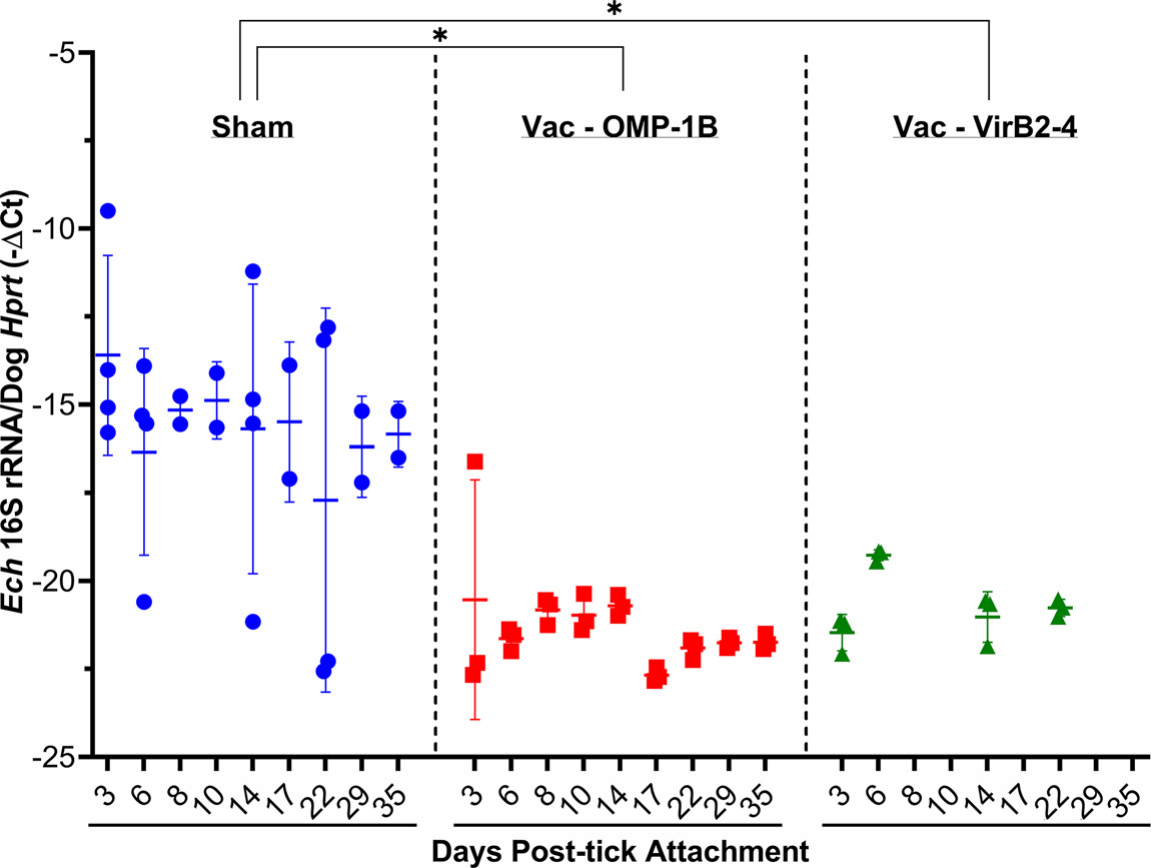
Vaccination of dogs with rOMP-1B or rVirB2-4 prevents tick transmission of *E. chaffeensis*. *E. chaffeensis* (*Ech*) load in peripheral blood from rOMP-1B- and VirB2-4-vaccinated and sham-vaccinated dogs after attachment of *E. chaffeensis-*infected ticks (RT-qPCR). −Δ*C_T_* (comparative cycle threshold): − (*C_T_* value of *E. chaffeensis* 16S rRNA −*C_T_* value of dog *HPRT* mRNA). *C_T_* values of >40 (undetectable) for *E. chaffeensis* 16S rRNA were capped as 45. Horizontal bars indicate mean values. Asterisks indicate significant differences between the rOMP-1B- or VirB2-4-vaccinated and sham-vaccinated dogs across all days postinfection (*P = *0.0039 for rOMP-1B and *P = *0.0047 for VirB2-4), as assessed by a mixed-effects model.

Substantial antibody titers specific to rOMP-1B and rVirB2-4 were attained for all vaccinated dogs but not for any of the sham-vaccinated dogs, as assessed by Western blotting and enzyme-linked immunosorbent assay (ELISA) throughout the study (Fig. 4A and B). The *E. chaffeensis* burden in the blood was monitored post-tick attachment by RT-qPCR (Fig. 5). At day 3 post-tick attachment, *E. chaffeensis* was detectable in blood from all four sham-vaccinated dogs but undetectable in blood from two of three OMP-1B-vaccinated and all three VirB2-4-vaccinated dogs (Fig. 5). After day 6 post-tick attachment, *E. chaffeensis* was undetectable in all six vaccinated dogs for the experiment duration (day 22 to 35 after tick attachment), whereas sham-vaccinated dogs were persistently infected from day 3 post-tick attachment (Fig. 5). Differences in bacterial 16S rRNA (normalized by the dog blood cell *HPRT1* [hypoxanthine phosphoribosyltransferase 1] mRNA) among the sham-vaccinated dogs (four dogs) and the dogs vaccinated with rOMP-1B (three dogs) or rVirB2-4 (three dogs) across all days post-tick attachment were statistically significant by mixed-effects model (47) (*P = *0.0039 for rOMP-1B, *P = *0.0047 for rVirB2-4) (Fig. 5). No significant clinical signs or abnormalities in blood cell counts or chemistry were detected in any of the ten dogs throughout the experiment.

### rVirB2-4 vaccination induces a significant IFN-γ response in dogs upon *E. chaffeensis*-infected tick challenge.

**FIG 6:**
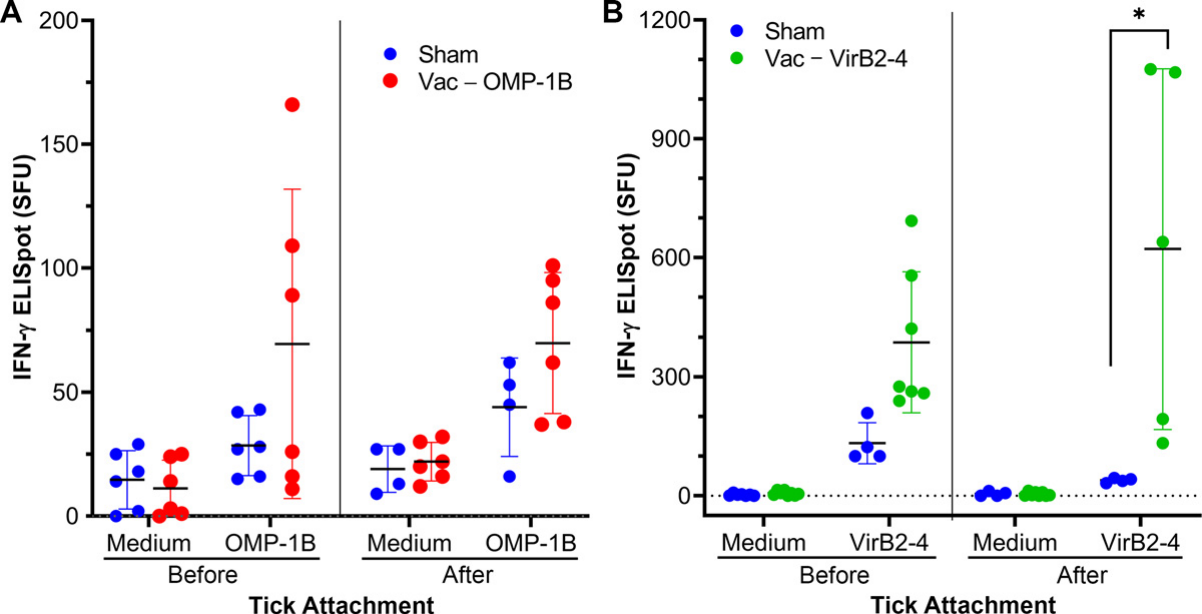
Vaccination of dogs with rVirB2-4, but not with rOMP-1B, induces a IFN-γ response to *E. chaffeensis* challenge. Peripheral blood mononuclear leukocytes (PBMCs) were isolated from three dogs vaccinated with rOMP-1B (A) and three dogs vaccinated with rVirB2-4 (B) and a total of four sham-vaccinated dogs at 1 (A) and 12 (B) days before and 7 days (A and B) after attachment of *E. chaffeensis*-infected ticks. PBMCs were incubated with medium (negative control), rOMP-1B (A), or rVirB2-4 (B) in triplicates or duplicates. SFU, spot-forming units. Differences in SFU between rOMP-1B- or rVirB2-4-vaccinated and sham-vaccinated dogs were assessed using a negative binomial mixed model. *, *P < *0.05.

IFN-γ is a robust indicator of a protective, cell-mediated immune response to *E. chaffeensis* infection *in vitro* and *in vivo* (in mice and dogs) (22, 48–51). Thus, the production of IFN-γ by live peripheral blood mononuclear leukocytes (PBMCs) was measured by ELISpot assay. The ELISpot assay allows the direct quantification of cells which secrete individual cytokines *ex vivo* based on IFN-γ capture ELISA. Furthermore, the sensitivity of ELISpot assay for cytokine detection in culture supernatants is 10- to 200-times greater than that of a traditional ELISA (52). A canine IFN-γ ELISpot assay revealed that peripheral PBMCs freshly collected from rOMP-1B-vaccinated dogs did not respond significantly to rOMP-1B by secreting IFN-γ at 1 week before and 1 week after attachment of infected ticks (Fig. 6A). However, PBMCs from rVirB2-4-vaccinated dogs significantly responded to rVirB2-4 stimulation at 1 week after attachment of infected ticks (Fig. 6B). PBMCs from sham-vaccinated dogs did not secrete substantial IFN-γ in response to rOMP-1B or rVirB2-4 stimulation at 1 week before and 1 week after attachment of infected ticks (Fig. 6A and B).

**FIG 7:**
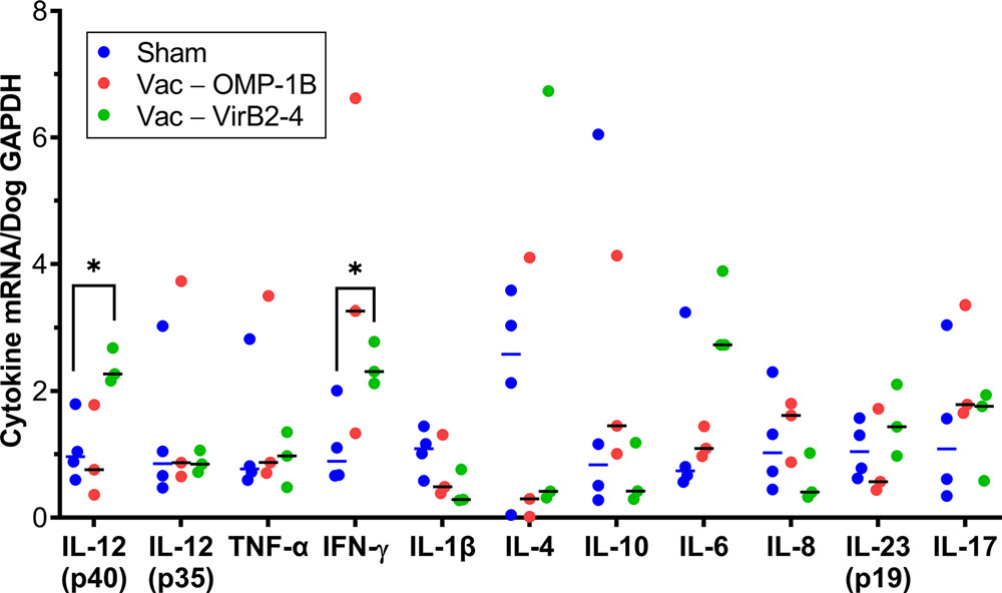
Expression of IL-12 and IFN-γ is upregulated in rVirB2-4-vaccinated and then challenged dogs. Expression of selected cytokine mRNAs normalized by dog GAPDH mRNA in blood samples from three rOMP-1B- and three rVirB2-4-vaccinated dogs and four sham-vaccinated dogs on day 7 after the attachment of *E. chaffeensis*-infected ticks (RT-qPCR). *, *P < *0.05 based on an unpaired two-tailed Student’s *t* test. Horizontal bars indicate mean values.

Cytokine mRNA expression by PBMCs was tested 1 week after *E. chaffeensis*-infected tick challenge in rOMP-1B- or rVirB2-4-vaccinated and sham-vaccinated dogs using previously published primers and methods (53). The resulting data were normalized by canine *GAPDH* (glyceraldehyde 3-phosphate dehydrogenase) mRNA using RT-qPCR. rVirB2-4-vaccinated dogs showed significantly higher expression of IL-12 (p40) and IFN-γ than did sham-vaccinated dogs, whereas rOMP-1B-vaccinated dogs did not show this difference (Fig. 7). Tick challenge after either rVirB2-4 or rOMP-1B vaccination did not induce any additional proinflammatory or immunostimulatory cytokine/chemokine response (mRNA for IL-8, IL-23, IL-6, IL-17, TNF-α, or IL-1β) as compared with sham-vaccinated dogs which subsequently underwent tick challenge (Fig. 7). Immunosuppressive cytokine (IL-4, IL-10) responses were not significantly different among rOMP-1B-, rVirB2-4-, and sham-vaccinated dogs upon challenge with *E. chaffeensis*-infected ticks (Fig. 7).

### rOMP-1B vaccination of dogs neutralizes *E. chaffeensis* infection in transmission-fed ticks.

**FIG 8:**
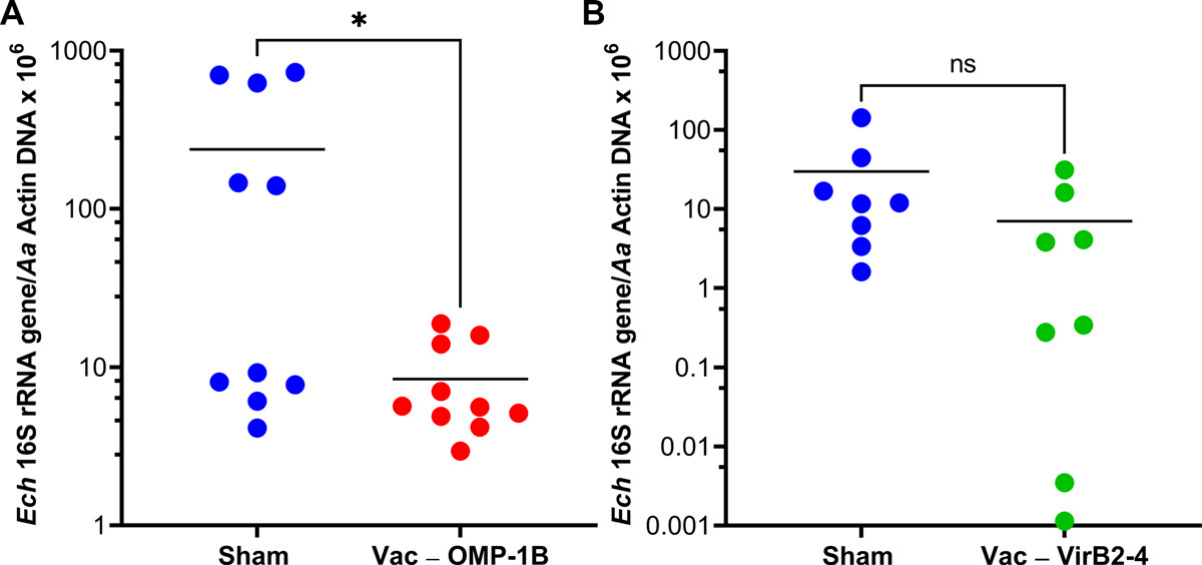
OMP-1B vaccination of dogs reduces *E. chaffeensis* infection in transmission-fed ticks. (A and B) Eight to ten female ticks each were randomly pulled off from four sham-vaccinated, three rOMP-1B-vaccinated (A), and three rVirB2-4-vaccinated (B) dogs at 10 days after tick infestation. DNA was isolated from each tick for assessment of *E. chaffeensis* (*Ech*) 16S rRNA and *A. americanum* (*Aa*) actin DNA by a qPCR assay. Actin levels were used to normalize the 16S rRNA levels. *, *P < *0.05 based on an unpaired two-tailed Student’s *t* test; ns, not significant. Horizontal bars indicate mean values.

Two sequential rOMP-1 and rVirB2-4 vaccination and tick challenge experiments were performed with two different batches of *E. chaffeensis*-infected ticks as previously described to ensure that they were freshly molted. qPCR revealed that the level of *E. chaffeensis* 16S rRNA gene (reflecting *E. chaffeensis* abundance) relative to *A. americanum actin* DNA in female ticks which fed on OMP-1B-vaccinated dogs was significantly lower than that in female ticks which fed on sham-vaccinated dogs (Fig. 8). Thus, the feeding of ticks on dog blood containing antibodies against OMP-1B appeared to reduce *E. chaffeensis* replication in infected adult ticks. In contrast, due to the high variability in *E. chaffeensis* abundance, there was no significant statistical difference between the female ticks fed on rVirB2-4-vaccinated dogs and those fed on sham-vaccinated dogs, although there was a clear trend toward lower infection rates in the rVirB2-4 group (Fig. 8).

## DISCUSSION

In this study, two animal models were used: mouse-intraperitoneal inoculation and dog-tick transmission models. Although the mouse infection model is unnatural, most vaccine studies on vector-borne obligatory intracellular bacteria have been carried out in mice challenged by needle inoculation (54) and almost all *Ehrlichia* pathogenesis and immunologic research used this model to derive immunologic or other signatures of responses (55–58). Dog-tick transmission is the natural mode of transmission. However, only a handful studies so far have used dogs for *E. chaffeensis* vaccine and/or challenge studies (22, 28, 51, 59, 60). Even fewer studies have used a tick transmission model. Because routes of infection profoundly influence immune responses and infection (61, 62), it is necessary to include a tick transmission model to improve vaccine design and regimens. So far, only two vaccine candidates, live attenuated *Ehrlichia* and EtpE-C subunit vaccines (22, 51), have been tested in a dog-tick transmission model. Neither vaccine prevented *Ehrlichia* transmission to dogs upon infected tick challenge: dogs were initially infected, but both vaccines accelerated clearance of *Ehrlichia* at 20 to 30 days after tick attachment in 3/3 dogs (51) and in 5/5 dogs (22). One new insight generated from the OMP-1B and VirB2 vaccine study is the absence of infection (undetectable by 16S rRNA gene-based qPCR) at much earlier post tick-challenge time points of 3 to 6 days, showing more effective blockade of tick transmission of *E. chaffeensis* than previous vaccines. Immune protection with rEtpE-C, rVirB2-4, and rOMP-1B vaccines seems to occur at different stages or processes of *E. chaffeensis* tick transmission and infection of dogs; therefore, a combination of these vaccines may provide better protection. Notably, compared with the strong immune responses induced in dogs by each of these three subunit vaccines (EtpE-C, VirB2-4, and OMP-1B), immune responses to these antigens were absent in sham-vaccinated dogs upon challenge with the infected ticks (based on poor antibody and IFN-γ responses). This may be due in part to the large variety of immunomodulatory substances which ticks inoculate into animals to incapacitate the host defense response to facilitate tick feeding and pathogen transmission (63, 64). Considering this, vaccines against tick-borne pathogens likely need to overcome these tick-derived immunosuppressive factors.

From an evolutionary point of view, dogs are more closely related to humans than mice (65) and share more similar physiological and immunobiological traits with humans. Immune responses induced by different mRNA vaccines in mouse and nonhuman primate models versus humans have shown that the CD8^+^ T cell response is much more apparent in mice, whereas it is often not detectable in monkeys or humans (66). Importantly, differences in the pathophysiology of an infection can underlie discrepancies among immune responses in animal models and humans, and consequently affect vaccine efficacy. Mice are resistant but dogs are inherently susceptible to *E. chaffeensis* infection. Thus, use of the mouse model may overidentify effective vaccine candidates or fail to identify important vaccine candidates such as in-tick neutralizing vaccine candidates, underscoring the importance of the dog-tick transmission model.

Identifying robust correlates of protection helps predictions of vaccine efficacy. However, except for antibodies and IFN-γ (23, 25–27, 48), protective immunity against *E. chaffeensis* is currently unknown. IFN-γ is upregulated in OMP-19 vaccine-induced protection of mice from mouse-pathogenic species of *Ehrlichia* (58). A correlation of IFN-γ upregulation and inhibition of *E. chaffeensis* was shown in tick-dog transmission models in previous studies with live attenuated *E. chaffeensis* vaccine (51) or rEtpE-C vaccine (22), and in this study with rVirB2-4 vaccine. The latter result is consistent with a previous study of Anaplasma marginale VirB2, which stimulates both antibody and T-cell responses in immunized cattle; epitope mapping showed T-cell epitopes in the conserved central region of VirB2 (67). These observations concur that IFN-γ-mediated immunity is a robust correlate of protection against *E. chaffeensis* infection. Understanding mechanisms of protective immunity is important to vaccine development. Previous studies showed mechanisms of IFN-γ-mediated inhibition of *E. chaffeensis* in human monocytes and macrophages are down-regulation of transferrin receptor, and sequestration of intracellular iron from *E. chaffeensis* *in vitro* (48, 68). Although this study did not find consistent correlations between other cytokines and the subunit vaccine efficacy, a correlation between a live attenuated vaccine's efficacy and IL-17 secretion by peripheral blood leukocytes has been reported in dogs (51).

By using tick-transmission model, insights into a new correlate of protection is generated: in-tick neutralization. To the best of our knowledge, OMP-1B is the first vaccine candidate that prevents replication of obligatory intracellular bacteria in ticks. The mechanisms of in-tick neutralization of *E. chaffeensis* remain to be investigated. The immunoglobulins consumed by ticks in a blood meal are specifically taken up across the midgut into the hemolymph by receptor-mediated endocytosis and are subsequently detected in the salivary gland (69–72). Thus, it is possible that dog antibodies against OMP-1B in the blood meal were taken up across the tick midgut and blocked the spread of *E. chaffeensis* among tick cells in the hemolymph and the salivary glands.

Antibody-mediated feedback regulation—regulation of the immune response to the antigen by the preexisting antibodies specific to the antigen—can result in >1,000-fold enhancement due to enhancement of antibody and CD4^+^ T-cell responses by activating FcγR, probably via increased antigen presentation by dendritic cells or >99% suppression of the specific antibody response due to epitope masking (73). Therefore, the standard booster interval is 2 to 3 weeks for mice (74) and the recommended vaccine interval is 2 to 4 weeks apart for initial adult canine vaccination (75). In the recent report of Lyme disease vaccine efficacy study in dogs, 3-week interval is used between two vaccinations (76). While the present study used the same booster intervals for both mouse and dog experiments, a longer vaccination interval (>3 weeks) merits consideration for future studies on immune responses to the vaccines in dogs. A Rickettsia rickettsii subunit (combination of Adr2 and OmpB-4) vaccine and its efficacy were tested in mice with booster immunization at 2 weeks after primary immunization (77) and in dogs with booster immunization at 4 weeks after primary immunization (78). Although several other experimental conditions are different between the two reports, mice seem to develop antibodies to the subunit vaccine more quickly than dogs following the primary immunization, and mice were protected upon R. rickettsii challenge whereas dogs were not. However, the present study did not find significant differences between mice and dogs.

Dogs and deer are the only experimental animal models currently available for tick-mediated transmission of *E. chaffeensis* (28, 42). To date, only the Arkansas strain has been used in deer (79), and the Arkansas and St. Vincent strains have been used for *E. chaffeensis* infection studies in dogs (28, 51, 59). Similar to what we observed in the present study, these strains do not cause acute severe disease in deer or dogs (28, 42, 51, 59, 60, 80, 81). Our study showed that mice can be used to screen vaccine candidates prior to tick transmission studies using dogs or deer, with the caveat that mice clear the *E. chaffeensis* infection within 10 days (23, 82). Thus, there is a need for a small laboratory animal that can serve as a tick-transmission disease model with *Ehrlichia* species. In this sense, *E. muris* subsp. *eauclairensis* (83) and *E. japonica* (84, 85) would provide excellent mouse models for tick vector transmission of monocytic ehrlichiosis. Although dogs are not currently considered major reservoirs of *E. chaffeensis* infection, naive ticks can acquire *E. chaffeensis* from infected dogs and subsequently transmit it upon biting naive dogs (28). Thus, infected dogs can serve as competent reservoirs of *E. chaffeensis*. Therefore, an OMP-1B- or VirB2-4-based vaccine is applicable for both humans and dogs to prevent the spread of *E. chaffeensis* infection to humans.

## MATERIALS AND METHODS

### Ethics statements.

All animal experiments were performed in accordance with the Ohio State University Institutional Animal Care and Use Committee guidelines and approved e-protocols. The university program has full continued accreditation by the Association for Assessment and Accreditation of Laboratory Animal Care International (AAALAC-I) under no. 000028 and has a Public Health Services assurance renewal no. A3261-01. The program is licensed by the U.S. Department of Agriculture no. 1-R-014 and is in full compliance with Animal Welfare Regulations.

### Preparation of *E. chaffeensis* cultures and host cell-free *E. chaffeensis*.

The canine macrophage cell line DH82 was used for culturing *E. chaffeensis* Arkansas (86) in Dulbecco’s minimal essential medium (DMEM; Mediatech, Manassas, VA) supplemented with 5% fetal bovine serum and 2 mM l-glutamine at 37°C in 5% CO_2_/95% air in a humidified atmosphere as previously described (48). Host cell-free *E. chaffeensis* was prepared as previously described (38). *E. chaffeensis* was also cultured in the tick cell line ISE6 as previously described (22).

### Cloning and purification of recombinant proteins.

*E. chaffeensis omp-1B* (ECH1136, GenBank accession no. WP_011453030) and *virB2-4* (ECH1042, GenBank accession no. WP_011452967.1) newly cloned in pET-33b (+) vector, and previously cloned *etpE-C* (C terminus of EtpE; ECH1038 GenBank accession no. WP_011452964.1) (18), were expressed in E. coli BL21(DE3) (Novagen; Madison, WI) induced with 0.5 mM isopropyl-β-d-thiogalactopyranoside (Gold Bio Technology, St. Louis, MO) at 30°C for 5 h. Both rEtpE-C and rOMP-1B were purified from the insoluble inclusions, dissolved in 6 M guanidine hydrochloride, and loaded onto a Poly-Prep chromatography column (Bio-Rad Inc. Hercules, CA) containing HisPur Cobalt Resin (Thermo Fisher Scientific, Waltham, MA). The column was washed with 10 mM imidazole in 8 M urea, and bound proteins were eluted with 250 mM imidazole in 8 M urea. rVirB2-4 was solubilized with 1 mM dithiothreitol (MilliporeSigma, Burlington, MA), 0.25% Na-deoxycholate (MilliporeSigma), 0.05% CHAPS (Bio-Rad) in sodium phosphate buffer (50 mM sodium phosphate [pH 7.4], 0.3 M NaCl, 1 mM phenylmethylsulfonyl fluoride). The purified proteins were run on a 10% to 12% SDS-polyacrylamide gel along with serial dilutions of known bovine serum albumin (BSA) standard for protein quantification. The SDS-polyacrylamide gel was stained with GelCode Blue Reagent (Thermo Fisher Scientific), destained with water, and imaged with the Amersham AI680QC gel documentation system (Cytiva, Marlborough, MA). The purified rOMP-1B or rVirB2-4 proteins were used for ISCOM vaccine preparation as previously described (22, 87). For mouse immunization, the relevant protein bands on the gels were individually excised with sterile scalpel blades and homogenized in a Dounce homogenizer in PBS (8 mM Na_2_HPO_4_, 1.47 mM KH_2_PO_4_, 2.67 mM KCl, 137.9 mM NaCl [pH 7.4]).

### Mouse immunization and challenge with *E. chaffeensis*.

Male mice (3 to 4 weeks old; ICR, Envigo, IN) each received a subcutaneous injection at two sites at the base of the neck with 50 to 100 μL of the homogenized gel containing 50 μg of (i) rEtpE-C, (ii) rVirB2-4, or (iii) rOMP-1B, or (iv) homogenized gel alone, each in combination with 10 μg Quil A (InvivoGen, San Diego, CA). Each mouse was injected a total of three times at 14-day intervals (5 mice/group). Blood samples were collected prior to immunization, at 6 days after the third immunization, and at euthanasia, and were used to determine antibody titers. *E. chaffeensis* challenge was performed 14 days after the last immunization via intraperitoneal inoculation with infected DH82 cells (>90% cells infected; 6 × 10^5^ cells/mouse). DNA was extracted from blood samples using a QIAamp DNA blood kit (Qiagen, Germantown, MD) and subjected to qPCR using *E. chaffeensis* 16S rDNA and primers specific for the mouse glyceraldehyde 3-phosphate dehydrogenase (GAPDH) gene (Table S1). RNA was extracted from the spleen and subjected to RT-qPCR using primers specific for mouse cytokines (88).

10.1128/mbio.02140-22.1 TABLE S1 Primers used for qPCR and RT-qPCR. Download Table S1, DOCX file, 0.03 MB. Copyright © 2022 Budachetri et al. 2022 Budachetri et al. https://creativecommons.org/licenses/by/4.0/ This content is distributed under the terms of the Creative Commons Attribution 4.0 International license.

### ELISA and Western blot analysis.

The wells of a 96-well flat-bottom microtiter plate (Nunc MaxiSorp, MilliporeSigma) were coated with 1 or 0.5 μg each of rEtpE-C, rOMP-1B, rVirB2-4, or BSA and ELISA analyses of sera from sham-vaccinated or vaccinated mice or dogs were performed as previously described (22). Western blot analysis using rOMP-1B or rVirB2-4 as antigens was performed as previously described (22).

### Infection of ticks with *E. chaffeensis* and analysis of ticks.

Freshly engorged nymphal *A. americanum* ticks were injected with host cell-free *E. chaffeensis* freshly isolated from infected DH82 cells (7 × 10^8^ to 10 × 10^8^ *Ehrlichia* per 2 to 4 μL, as assessed by qPCR) and maintained in an incubator as previously described (22). Randomly selected adult male and female ticks were tested for *E. chaffeensis* infection and OMP-1B and VirB2-4 expression by RT-qPCR prior to dog challenge. Ticks which had fed on dogs were pulled off the dogs after 10 to 13 days post-infestation, and *E. chaffeensis* infection was determined in females by qPCR.

### Vaccination of dogs and challenge with infected ticks.

Ten SPF beagle dogs (1 to 2 years old) were purchased from Covance (five dogs), Marshall Farm (two dogs), and Envigo (three dogs) and were housed in University Lab Animal Resources, College of Veterinary Medicine, Ohio State University. Three dogs each were vaccinated with rOMP-1B-ISCOM and rVirB2-4-ISCOM, and four dogs were injected with PBS-ISCOM subcutaneously to the subscapularis region on both sides (0.5 mL each side); each dog received three injections, with each separated by a 2-week interval.

For the challenge, two sites in the subscapularis area of each dog were shaved and washed, and a 2-in section of tubular stockinette cotton roll (Medichoice, Mechanicsville, VA) was glued to each site with Animal ID tag cement (Nasco, Fort Atkinson, WI) (two stockinette feeding chambers per dog). *E. chaffeensis*-infected ticks (20 females, 10 males) were placed in each feeding chamber and allowed to feed until they started dropping off (10 to 13 days). Both male and female ticks were included in each chamber to promote feeding on blood and *E. chaffeensis* transmission because the males must feed to produce spermatophores and the females must feed to produce eggs (44). Clinical signs and rectal temperatures of the dogs were monitored daily, and blood parameters (complete blood count with white blood cell differential and serum chemistry) were analyzed before the first vaccination, after the third vaccination, and at 1 and 3 weeks after infected tick challenge. Blood samples (4 to 5 mL) were collected from the saphenous vein at 3 to 35 days after challenge. An aliquot of the whole blood (400 μL) was saved for DNA isolation, and buffy coats from 3 mL of blood were used for RNA isolation; plasma was saved for ELISA or Western blotting to determine the titers of antibodies against OMP-1B and VirB2-4.

### Isolation of dog PBMCs and ELISpot assay.

PBMCs were isolated using Ficoll-Paque Plus (GE Healthcare, Piscataway, NJ) as previously described (22). The ELISpot assay was performed using the Canine IFN-γ ELISpot kit (R&D Systems, Minneapolis, MN). Briefly, 5 × 10^5^ PBMCs/well were plated in a 96-well plate coated with canine IFN-γ. The cells were stimulated with rOMP-1B (1 μg), rVirB2-4 (1 μg), or culture medium (as a negative control) in triplicate or duplicate wells per dog. The immunopositivity of spots was assessed with an ImmunoSpot S6 Analyzer (Cellular Technology, Cleveland, OH). The data were analyzed by a negative binomial mixed model (22, 89).

### RT-qPCR and qPCR.

Individual ticks (male or female) after molting, mouse spleen, *E. chaffeensis*-infected ISE6 tick cells, and buffy coats collected from dog blood were used to isolate total RNA as described elsewhere (22). Mouse blood samples and individual female ticks which had been removed from a dog were used to isolate DNA. RT-qPCR and qPCR were performed with cDNA or DNA, respectively, using gene-specific primers (Table S1) as described elsewhere (22). Expression ratios of target genes relative to a reference gene (*E. chaffeensis* 16S rRNA/tick actin; or mouse GADPH or dog HPRT or GAPDH, OMP-1B, or VirB2-4/16S rRNA; and mouse or dog cytokines/GAPDH) were estimated by the standard method (90) or as previously described (22).

### Statistical analysis.

Statistical analyses were performed with an unpaired two-tailed Student’s *t* test or analysis of variance, as applicable. For the *E. chaffeensis* 16S rRNA cycle threshold (*C_T_*) data, analysis was performed with the mixed-effects model using R package version 3.1-148 (47, 91). *C_T_* values of >40 were capped at 45. This is essentially conservative because the true *C_T_* value may be higher than 45. *P < *0.05 was considered significant. Differences in spot-forming units between rOMP-1B- or rVirB2-4-vaccinated and sham-vaccinated dogs were assessed using the lme4 package (89) of a negative binomial generalized linear mixed model. *P < *0.05 was considered significant. All graphs and other statistical calculations were prepared with Prism 8 software (GraphPad, San Diego, CA).
