# Supplementary material for: Efficacy and Immune Correlates of OMP-1B and VirB2-4 Vaccines for Protection of Dogs from Tick Transmission of Ehrlichia chaffeensis
Source: mBio. 2022 Nov 7;13(6):e02140-22. doi: 10.1128/mbio.02140-22 (PMC9765013; doi:10.1128/mbio.02140-22)
Supplement: TABLE S1 [file mbio.02140-22-s0001.docx]

# SUPPLEMENTARY MATERIALS

# Table S1. Primers used for qPCR and RT-qPCR

| Primer name | Target gene  (accession number) | Primer sequence (5'-3') | Amplicon size | Reference |  |
| --- | --- | --- | --- | --- | --- |
| KB019 | *E. chaffeensis*  Omp-1B (ECH1136) ^1^ | GTACCCATGGATCCTGTAACTTCAAATGATACAG | 777 | This study |  |
| KB020 |  | TACGGCGGCCGCGAAGGTGAACCTTACTCCAACTTC |  |  |  |
| VB2.4F | *E. chaffeensis*  VirB2-4 (ECH1042) ^2^ | GGGGGATCCGAACGAGCAAGCTGGTACTGGTG | 291 | This study |  |
| VB2.4R |  | AAACTCGAGTCACTTAACCTGGCTACTTGGC |  |  |  |
| KM0125 | *E. chaffeensis*  16S rRNA (NR_074500.2) | CGGGGGAAAGATTTATCGCTATTA | 323 | (1) |  |
| KM0126 |  | CGCTTGCCCCCTCCGTATTA |  |  |  |
| K9RT_ HprtF | Dog HPRT1  (NM_001003357.2) | CAGCCTTGGCGTCGTGATTAG | 195 | This study | |
| K9RT_ HprtR |  | TTGAGCACACAGAGGGCTAC |  |  |  |
| KB007 | *A. americanum* actin  (EZ000248.1) | CCGTGAGAAGATGACCCAAA | 123 | (2) |  |
| KB008 |  | CCGGAGTCGAGCACAATAC |  |  |  |
| KB009 | *I. scapularis* actin  (XM_002413517.1) | CTTGGAGGGAGACATCTTTGTG | 111 | (2) |  |
| KB010 |  | CGCTCCATGTCATTCCAATCT |  |  |  |
| KB011 | Human actin  (NM_001101.4) | AGAGCTACGAGCTGCCTGAC | 184 | (3) |  |
| KB012 |  | AGCACTGTGTTGGCGTACAG |  |  |  |
| HB001 | Mouse GAPDH  (NM_001289726.1) | GTTGTCTCCTGCGACTTCA | 184 | (4) |  |
| HB002 |  | GGTGGTCCAGGGTTTCTTA |  |  |  |
| KB025 | Dog GAPDH  (NM_001003142) | ATCACTGCCACCCAGAAGAC | 133 | (5) |  |
| KB026 |  | TCAGCTCAGGGATGACCTTG |  |  |  |
| KB027 | Dog IFN-γ  (NM_001003174) | GCGCAAGGCGATAAATGAAC | 82 | (5) |  |
| KB028 |  | CTGACTCCTTTTCCGCTTCC |  |  |  |
| KB029 | Dog IL-1β  (NM_001037971) | CAAGTCTCCCACCAGCTCTGTA | 81 | (5) |  |
| KB030 |  | GGGCTTCTTCAGCTTCTCCAA |  |  |  |
| KB031 | Dog IL-12 p40  (NM_001003292) | CAGCAGAGAGGGTCAGAGTGG | 109 | (5) |  |
| KB032 |  | ACGA CCTCGATGGGTAGGC |  |  |  |
| KB049 | IL-12 p35 (NM_001003293) | GTGCCTCAACCACTCCCAA | 101 | (5) |  |
| KB050 |  | CAATCTCTTCGGAAGTGCAGG |  |  |  |
| KB033 | Dog TNF-α  (NM_001003244) | TCTCGAACCCCAAGTGACAAG | 153 | (5) |  |
| KB034 |  | CAACCCATCTGACGGCACTA |  |  |  |
| KB021 | *E. chaffeensis*  Omp-1B (ECH1136) | ATACCGTATGCATGTGCAGGT | 124 | This study |  |
| KB022 |  | AAGCGGAAACTTCTGGTGTGA |  |  |  |
| KB023 | *E. chaffeensis*  VirB2-4 (ECH1042) | CCGTTGTGTTACTCAGGTGC | 315 | This study |  |
| KB024 |  | AACCTGGCTACTTGGCGTTA |  |  |  |
| KB039 | IL-4 (NM_001003159) | TCACCAGCACCTTTGTCCAC | 144 | (5) |  |
| KB040 |  | CGCTTGTGTTCTTTGGAGCA |  |  |  |
| KB041 | IL-17 (NM_0011658780) | GGAATCTGCACCGCAATGAGGAC | 148 | (5) |  |
| KB042 |  | CGCAGAACCAGGATCTCTTGCTGG |  |  |  |
| KB043 | IL-10 (NM_001003077) | CGGGAGGGTGAAGACTTTCT | 144 | (5) |  |
| KB044 |  | GGCATCACCTCCTCCAAGTA |  |  |  |
| KB045 | IL-6 (NM_001003301) | TTAAGTACATCCTCGGCAAAATCT | 86 | (5) |  |
| KB046 |  | CAGTGCCTCTTTGCTGTCTTCA |  |  |  |
| KB047 | IL-8/CXCL8 (NM_001003200) | CTCTCTGTGAAGCTGCAGTTCTG | 81 | (5) |  |
| KB048 |  | GGAAAGGTGTGGAGTGTGTTTTT |  |  |  |
| KB051 | IL-23 p19 (XM_538231) | CAAGGGGAGAAAAACAGCAG | 79 | (5) |  |
| KB052 |  | TGCTGTCCGTTCTGTGAGTC |  |  |  |

## Note:

^1^ Primers for amplifying *E. chaffeensis omp-1B* gene at 76 – 849 bp (with signal peptide sequences and stop codon removed) and cloning into pET33b(+) expression vector at NcoI and NotI sites (underlined sequences).

2 Primers for amplifying *E. chaffeensis virB2-4* gene at 82 – 372 bp (with signal peptide sequences and stop codon removed) and cloning into pET33b(+) expression vector at BamHI and XhoI sites (underlined sequences).

# REFERENCES:

1. Wang X, Rikihisa Y, Lai TH, Kumagai Y, Zhi N, Reed SM. 2004. Rapid sequential changeover of expressed *p44* genes during the acute phase of *Anaplasma phagocytophilum* infection in horses. Infect Immun 72:6852-9.

2. Budachetri K, Teymournejad O, Lin M, Yan Q, Mestres-Villanueva M, Brock GN, Rikihisa Y. 2020. An Entry-Triggering Protein of Ehrlichia Is a New Vaccine Candidate against Tick-Borne Human Monocytic Ehrlichiosis. mBio 11.

3. Song Y, Zuo Y. 2014. Occurrence of HHIP gene CpG island methylation in gastric cancer. Oncol Lett 8:2340-2344.

4. Bekebrede H, Lin M, Teymournejad O, Rikihisa Y. 2020. Discovery of in vivo Virulence Genes of Obligatory Intracellular Bacteria by Random Mutagenesis. Front Cell Infect Microbiol 10:2.

5. Tamura Y, Ohta H, Yokoyama N, Lim SY, Osuga T, Morishita K, Nakamura K, Yamasaki M, Takiguchi M. 2014. Evaluation of selected cytokine gene expression in colonic mucosa from dogs with idiopathic lymphocytic-plasmacytic colitis. J Vet Med Sci 76:1407-10.
